# Newborns’ neural processing of native vowels reveals directional asymmetries

**DOI:** 10.1016/j.dcn.2021.101023

**Published:** 2021-10-20

**Authors:** Kateřina Chládková, Josef Urbanec, Sylva Skálová, Jan Kremláček

**Affiliations:** a Institute of Czech Language and Theory of Communication, Faculty of Arts, Charles University, Nám. Jana Palacha 2, 116 38 Praha, Czechia; b Institute of Psychology, Czech Academy of Sciences, Hybernská 8, 110 00 Praha, Czechia; c Department of Pathological Physiology, Faculty of Medicine in Hradec Králové, Charles University, Šimkova 870, 500 03 Hradec Králové, Czechia; d Paediatrics Department, Havlíčkův Brod Hospital, Husova 2624, 580 01 Havlíčkův Brod, Czechia; e Paediatrics Department of University Hospital, Sokolská 581, 500 05 Hradec Králové, Czechia; f Department of Medical Biophysics, Medical faculty in Hradec Králové, Charles University, Šimkova 870, 500 03 Hradec Králové, Czechia

**Keywords:** Language development, Prenatal learning, Speech sound acquisition, Category formation, Newborn ERP

## Abstract

Prenatal learning of speech rhythm and melody is well documented. Much less is known about the earliest acquisition of segmental speech categories. We tested whether newborn infants perceive native vowels, but not nonspeech sounds, through some existing (proto-)categories, and whether they do so more robustly for some vowels than for others. Sensory event-related potentials (ERP), and mismatch responses (MMR), were obtained from 104 neonates acquiring Czech. The ERPs elicited by vowels were larger than the ERPs to nonspeech sounds, and reflected the differences between the individual vowel categories. The MMRs to changes in vowels but not in nonspeech sounds revealed left-lateralized asymmetrical processing patterns: a change from a focal [a] to a nonfocal [ɛ], and the change from short [ɛ] to long [ɛ:] elicited more negative MMR responses than reverse changes. Contrary to predictions, we did not find evidence of a developmental advantage for vowel length contrasts (supposedly most readily available in utero) over vowel quality contrasts (supposedly less salient in utero). An explanation for these asymmetries in terms of differential degree of prior phonetic warping of speech sounds is proposed. Future studies with newborns with different language backgrounds should test whether the prenatal learning scenario proposed here is plausible.

## Highlights

• Newborns’ processing of native vowels and comparable nonspeech sounds differ.
• Durational and spectral differences in stimuli were more clearly reflected by the ERPs to vowels, compared to tone complexes.
• Directional asymmetries were detected in the mismatch responses to vowel deviants.
• In the left hemisphere, a change in vowels from focal to nonfocal and from short to long resulted in a more negative MMR.
• The findings may be explained by phonetic learning prior to the 3rd day after birth.

## 1 Introduction

Humans learn about their mother’s voice, language, and frequently recited rhymes while still in the womb (Mehler et al., 1988, DeCasper et al., 1994, Kisilevsky et al., 2009). These early linguistic abilities have been attributed to fetal sensitivity to language prosody, that is, its rhythm and intonation (Moon et al., 1993, Granier‐Deferre et al., 2011, Abboub et al., 2016). Newborn cortices indeed show specialization for listening to streams of speech over non-speech, and process native-language speech differently from non-native speech (May et al., 2018, Sato et al., 2012).

Besides prosody, languages differ vastly in the speech segments that they employ to construct and contrast words: for instance, British English contrasts 44 segmental categories, while Central Rotokas, a language spoken in Papua New Guinea, only has 11 (Maddieson, 1986). Unlike prosody, however, whose prenatal acquisition has been studied relatively widely, the earliest linguistic development of individual speech segments is less documented. The earliest stage of segmental speech sound processing and learning is examined in the present study. We ask whether newborn infants’ processing of speech sound contrasts displays any evidence of prior, i.e. prenatal, experience with those contrasts.

A review of existing literature suggests that the intrauterine linguistic development could comprise learning even of segmental properties of speech. Firstly, the speech signal *in utero* preserves some of the acoustic properties that cue segmental identity. Sounds’ spectral properties are relatively well preserved in the range up to ~1000 Hz with higher frequencies being progressively attenuated by about 6 dB/octave, although these values vary across studies (see Granier‐Deferre et al., 2011). The higher frequency range thus gets diminished while lower frequencies, including durational modulations, reach the fetus in a virtually unchanged form, or might even be perceptually strengthened (Richards et al., 1992). The preservation of low-frequency and durational characteristics then enables the fetus to hear and learn the language-specific intonational and rhythmic patterns (Querleu et al., 1988, Granier‐Deferre et al., 2011, Abboub et al., 2016). Crucially, not only rhythm but also some segmental categories of speech are cued by frequency information below ~1000 Hz and by duration, which leads to the hypothesis that the developing human could start acquiring segmental speech categories during the prenatal period.

A normally developing fetus is able to hear and process the encountered acoustic signal. At around 20 weeks of gestation, neuronal connections in the peripheral and central auditory system begin to be formed and tonotopic organization develops in the cochlea, and from about gestational week 28 in the temporal cortex (Graven and Browne, 2008). From at least the 35th gestational week fetuses perceptually discriminate tones with frequencies 250 Hz versus 500 Hz, and vowels [i] versus [a] embedded either in a [b_] or a [b_b_] context (Shahidullah and Hepper, 1994, Lecanuet et al., 1987). However, 36-week old fetuses do *not* discriminate the consonantal [da]-[ta] distinction (mainly distinguished by frication above 2000 Hz) although pre-term infants born at 29–32 weeks do discriminate a (different) consonantal contrast [ba]-[ga] (Weikum et al., 2012, Mahmoudzadeh et al., 2013). These studies suggest that some speech sounds, such as consonantal contrasts cued in a high-frequency range, may not be audible and/or discriminable *in utero* to the same extent as some vowels or tones are.

A handful of relatively recent studies indicate that fetuses can engage in the process of speech sound learning. Partanen et al. (2013) found that infants who received prenatal training with rare pitch and vowel quality variations in a frequently exposed pseudoword [tatata] had enhanced neural processing of pitch differences at birth as compared to a group of untrained infants. Specifically, infants were more sensitive to changes in vowel fundamental frequency (averaging around 170 Hz) if they were exposed to them prenatally (Partanen et al., 2013). Besides such prenatal controlled exposure, another study suggests that newborn speech sound perception may be influenced by natural language environment. Moon et al. (2013) showed that 1- to 4-days old American English and Swedish infants differ in how they behaviourally react to American English /i/ and Swedish /y/, acoustically differentiated in the low frequency range at about 250 Hz, as well as in the higher frequency range 2–3 kHz. Infants from either group were perceptually more sensitive to variants of the *non-*native vowel category (in line with language-specific categorical perception), meaning that they processed native and non-native vowels differently. A reanalysis of Moon et al.’s (2013) data reported by Zhao et al. (2011) further supports the role of native language exposure during prenatal development. The native-language effect seems to have been driven by those newborns who had an older sibling (4 years or younger) – and thus likely overheard infant-directed, i.e., exaggerated and affective, speech during their prenatal development – than in infants without such a sibling. Moon et al.’s (2013) data thus indicate that the learning of native vowel categories from exposure might start already before birth.

In sum, humans can hear and are capable of learning the speech sounds of their native language before birth. Since vowels are (prenatally) the most perceptually salient sounds, they are also the focus of the present study. Languages commonly contrast anywhere between 5 and 35 vowel categories, such that within the class of vowels one will likely find various patterns and onsets of learning. In some languages (e.g. Finnish, Japanese, or Czech), duration cues not only prosody but also segmental short-long vowel contrasts. Given the veridical transmission of the durational cues to the womb, as opposed to the modulations affecting vowel spectrum, one could hypothesize that in languages with contrastive vowel length, durationally-cued vowel categories will have a developmental advantage over spectrally-cued ones. To test that hypothesis, this study focuses on two types of vowel contrasts: one durational and one spectral.

We assess the neural processing of speech sounds in one-to-three days old infants, who had been exposed to a language that systematically differentiates vowels both by duration and by spectral quality (namely, Czech). The newborns are tested on their processing of durational and spectral changes in two sets of stimuli: speech and nonspeech. Both stimulus sets contain similar acoustic patterns but in different contexts – in one context these patterns occur in vowel stimuli that specify the native-language categorical contrasts /ɛ/-/a/ and /ɛ/-/ɛː/ and in the other context they occur in complex inharmonic tones that are not interpretable as speech.

To measure whether the newborns employ categorical ‘knowledge’ during stimulus processing, we assess their mismatch responses (MMR). The MMR is particularly suited as an index of higher perceptual processing because it quantifies the conflict between a prediction created on the basis of one stimulus and its violation caused by another stimulus (Näätänen, 2001, Winkler and Czigler, 2012). In infants and children, the MMR has been employed to assess the formation of language-specific speech sound representations (Cheour et al., 2002; Cheour et al., 1998, Nenonen et al., 2005). Initially in development, the size of the MMR seems mainly correlated with acoustic distance between speech stimuli, but as linguistic representations come to be formed, the categorical mental encoding overrides the acoustic distance effect and becomes the primary modulator of the MMR (Cheour et al., 1998). Besides its size, the polarity of the MMR to speech has been shown to reflect the developmental stage of an individual and/or of a particular linguistic contrast, where a negative deflection of the MMR characterizes a more mature response than a positive deflection (Maurer et al., 2003, Mueller et al., 2012, Thiede et al., 2019) and/or a contrast that is easier to discriminate (Peter et al., 2016). The MMR thus seems ideal means for uncovering the extent to which newborn infants employ prior experience with speech sounds when processing different types of stimuli.

With respect to our hypothesis of developmental advantage of vowel length over vowel quality, we can formulate predictions both about the strength and the polarity of the MMR. Firstly, we expect the MMR to changes in vowel duration to be more robust, i.e. of greater amplitude than the MMR to changes in vowel spectral quality. Regarding the polarity, vowel length changes could result in a negative-going MMR while vowel quality changes in a positive-going MMR.

Studies on perceptual discrimination of vowels, with both infants or adults, often report directional asymmetries. For instance, within the /i/-/ɛ/ contrast, young ‘pre-linguistic’ infants might be more sensitive to a change from /ɛ/ to /i/ than to a change from /i/ to /ɛ/ (Polka and Bohn, 2011). Peripheral vowels like /i/ or /a/ are characterized by stable articulatory-acoustic relations, as well as by a concentration of acoustic energy in a particular frequency range (i.e. focalization), while non-peripheral vowels like /ɛ/ are not: these differential phonetic properties have been argued to cause the asymmetries in infants’ vowel perception (Polka and Bohn, 2003, Polka and Bohn, 2011, Schwartz et al., 2005). Note however that not all studies with infants found such perceptual asymmetries (Wanrooij et al. 2014) and that adults may even display reverse asymmetries (Scharinger et al., 2011, Lahiri and Reetz, 2010). To account for the possibility that also newborn infants have a perceptual asymmetry, the present study employs a stimulation paradigm that allows to assess the MMR to changes in both directions within individual participants in a reasonable amount of time. No specific a priori predictions were formulated about the directional asymmetries, but they will be returned to in the Discussion.

Prior to analysing MMR, we will assess the newborns’ primary sensory responses (ERPs) to the different auditory stimuli. Physically different stimuli typically elicit different sensory ERPs, e.g. in adults the amplitude of the ERP approximately 100 ms after stimulus onset, the N1, is inversely related to vowel first formant (Scharinger et al. 2011). Since the infants tested here have normally developing hearing we predict that they will process the acoustic differences between the [ɛ] and [a]-like stimuli and between the short and long stimuli in both the speech and the nonspeech condition. Therefore, the ERPs elicited by [ɛ](-like) and [a](-like) and by short and long sounds are predicted be different.^1^

To summarize, the experiment reported here investigates whether the acquisition of native vowels is underway already before birth and whether durational contrasts have an early advantage over spectral contrasts. Given the loudness and *intrauterine* availability of at least some vowel cues, it is likely that normally developing infants will have already started the process of category formation for the vowels of their native language. Considering the absolute veridical transmission of acoustic duration and the gradual attenuation of frequency information, we predict that durationally-cued vowel categories are at birth acquired more robustly than spectrally-cued vowel categories. Possibly, one or both types of vowel contrasts may result in asymmetric patterns in the MMR with one direction of change causing a stronger MMR response than the other direction. If the effects that we predict for vowels (the advantage of vowel length over vowel quality and/or any directional asymmetries) are due to prior exposure to the sounds they should not be observed for non-linguistic stimuli.

## 2 Method

### 2.1 Stimuli

#### 2.1.1 Speech and non-speech segments

Speech stimuli were naturally produced, edited consonant-vowel (CV) syllables [fɛ] and [fa]. The vowel formants were stable throughout and representative of the Czech low-mid front /ɛ/ and low /a/, respectively. The first three formants (i.e. F1, F2, and F3) of [ɛ] in [fɛ] were 755 Hz, 1646 Hz, and 2710 Hz. The first three formants of [a] in [fa] were 864 Hz, 1287 Hz, and 2831 Hz. The vowels [ɛ] and [a] were extracted and their durations edited using PSOLA in Praat (Boersma and Weenink, 1992–2020). We made one [a] with a duration of 220 ms, and three [ɛ]’s, namely, 220 ms, 180 ms, and 360 ms. These durations fulfilled the following criteria: 220 ms was judged (by 3 expert phoneticians) as a typical duration of the mid and low short Czech vowels in an isolated CV syllable, 360 ms was representative of a long Czech vowel in a CV syllable that was not perceived as unnaturally exaggerated, and 180 ms was judged as sufficiently distinct from the long vowel, also based on the previously reported finding that short low and mid vowels are in Czech about half the duration of their long counterparts (Paillereau and Chládková, 2019). Note that in Czech both short and long vowels are legitimate in open syllables.

From a different recorded syllable [fɛ] we cut out the initial fricative [f], which had a duration of 150 ms, and spliced it onto the target [a] and [ɛ] vowels. The fricative [f] was thus identical across all four speech stimuli. Neither of the [f]+vowel monosyllables carries lexical or morphological content in Czech.

**Fig. 1:**
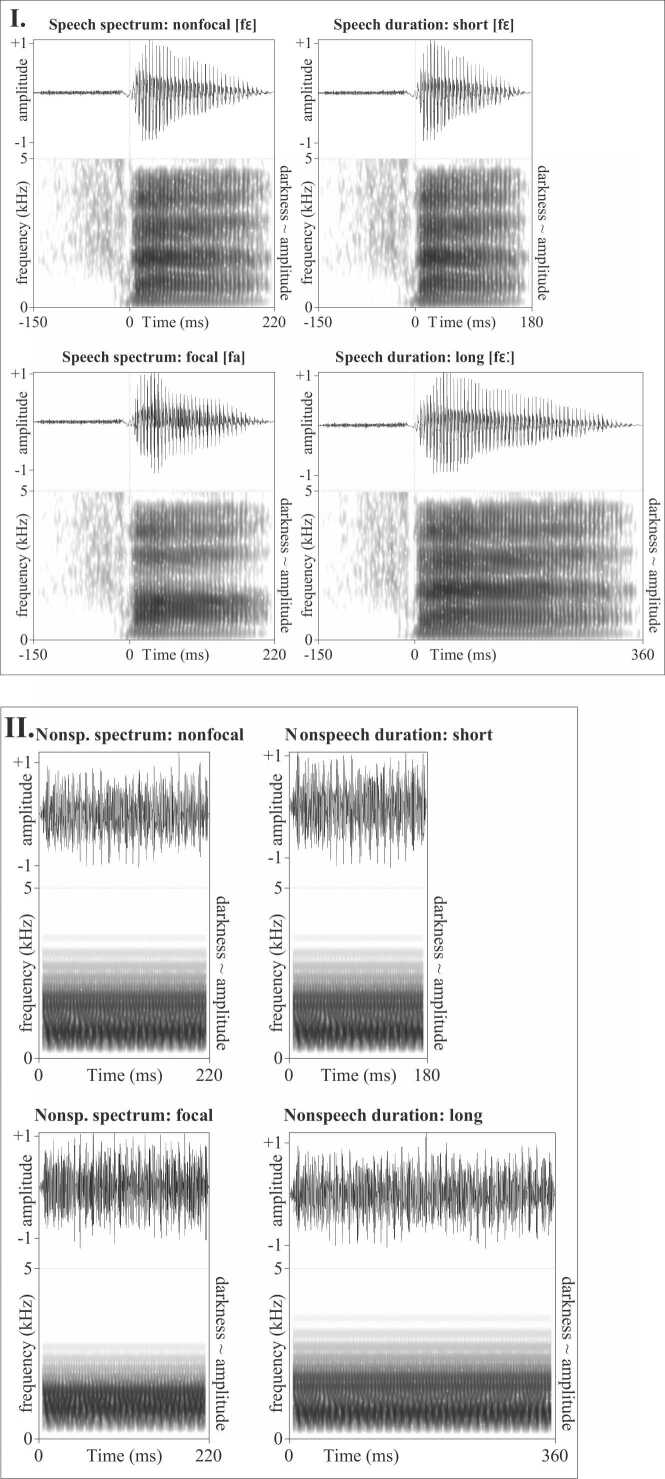
Oscillograms and spectrograms of the speech (I.) and nonspeech stimuli (II.). The depicted amplitude scale is relative, both the speech and nonspeech stimuli were presented at 67 dB SPL (as measured by a dummy head using infant earcouplers with fitted earplugs).

The four speech stimuli are visualized in Fig. 1, box I. The 220-ms [fɛ] and the 220-ms [fa] tested discrimination of a spectral contrast, which is why they are referred to as spectrally nonfocal and spectrally focal, respectively. The [a] in [fa] is focal because its first two formants (visible in the spectrograms of Fig. 1 as black horizontal bars) are close to one another (merging into a single black horizontal bar in the spectrogram); the [ɛ] in [fɛ] is termed as nonfocal, because its first and second formant are spread apart (and clearly visible as two separate horizontal bars in the spectrogram). The 180-ms [fɛ] and the 360-ms [fɛ] were used to test discrimination of a durational contrast, and are referred to as short and long, respectively. Average stimulus intensity was equated across all four syllables.

Nonspeech stimuli were inharmonic tone complexes with spectral and durational properties mimicking those of the vowels described above. Inharmonic tone complexes have a similar acoustic structure to vowels in that their source signal contains a series of exponentially spaced frequencies, and is filtered with vocal-tract like formants. At the same time, the inharmonic tone complexes are not confusable with vowels because their source-signal frequencies are spaced inharmonically (Goudbeek et al., 2009, Scharinger et al., 2014). The difference in language-likeness between the conditions was further strengthened by using CV syllables as the speech stimuli but isolated individual tone complexes as the non-speech stimuli.

The tone complexes in the present experiment had 15 inharmonically spaced frequency components, the first one at 500 Hz and every following being 1.15 times higher. The inharmonic source signal was filtered with three formants, namely, for the focal spectral condition with the formants of [a], for the nonfocal spectral condition and the short and long durational condition with the formants of [ɛ]. The tone complexes were acoustically somewhat simpler in spectral content than the vowels because they were filtered with 3 formants, while the vowels also had spectral content in higher frequencies (as can be seen in Fig. 1). Since monophthongal low vowels, such as the [ɛ] and [a] used here, are sufficiently differentiated by the first two formants (and F3 helps to normalize for talker variation, Monahan and Idsardi, 2010), the non-speech synthesis with F1, F2, and F3 was considered adequate for comparing the discrimination of vowel(-like) spectral quality across speech and non-speech. Durations of the nonspeech stimuli were identical to the durations of the vowels from the speech condition. The amplitude was ramped linearly over 5 ms at stimulus onset and offset (in contrast to the speech stimuli, the non-speech stimuli had a more uniform amplitude envelope, as seen in Fig. 1). Average sound intensity was equated across all the four nonspeech stimuli, as well as across speech and nonspeech.

The nonspeech stimuli are plotted in Fig. 1, box II. As in the speech condition, the [a]-like focal tone and the [ɛ]-like nonfocal 220-ms tone were used to test discrimination of spectral differences, and the 180-ms [ɛ]-like tone and the 360-ms [ɛ]-like tone were used to test discrimination of duration differences. The stimuli are the same as those used in Nudga et al., 2021 who measured MMN to vowel and nonspeech contrasts with Czech adults.

#### 2.1.2 Stimulus presentation

Stimuli were presented in a roving-standard paradigm (e.g. Haenschel et al., 2005). Four presentation blocks were created, one for each domain (speech and nonspeech) and dimension (spectrum and duration) combination. For speech spectrum, the paradigm started with 8 tokens of [fɛ] and continued with 100 trains of [fɛ] and [fa] each, alternating in series’ of 4–8 identical stimuli. The count of 4–8 was pseudorandom, fulfilling the condition that each count eventually occurred 20 times. The number of presented tokens was 608 for [fɛ], and 600 for [fa]; summing up to a total of 1208 stimuli in each block. Stimulus-onset asynchrony was 1.09 s. Total presentation time per block was 22 min. The blocks for speech duration were created in an identical way, alternating series’ of short [fɛ]s and the long [fɛː]s. Analogous presentations were made for nonspeech spectrum and nonspeech duration.

An individual infant was tested with either the two speech blocks, or the two nonspeech blocks. Stimulus domain thus varied between participants and dimension within participants, with the order of durational and spectral presentation counterbalanced between infants.

### 2.2 Participants

The participants were 104 full-term, healthy infants (16 additional infants were tested but excluded due to fussiness or noisy recording).^2^ Their physiological details are given in Table 1. All infants’ Apgar score (vitality index) at the 10th minute after birth was 10 (highest value), and all passed the neonatal hearing test. Physiological vaginal and uncomplicated caesarean births were included. All mothers were monolingual native speakers of Czech. The infants were judged as low-risk regarding developmental language or speech-related disorders (based on absence of symptoms in parents and siblings).

**Table 1:** Infant demographics per the between-subject condition, domain.

condition	*n* included (*n* tested)	*n* per sex	age at experiment:	birth weight:
			mean (range)	mean (range)
speech	54 (60)	30 F, 24 M	57 h (30 – 108)	3395 g (2720–4420)
nonspeech	50 (60)	25 F, 25 M	54 h (28 – 87)	3363 g (2620–4100)

### 2.3 Procedure

The experiment was approved by the ethics committee of the Faculty of Medicine and University Hospital in Hradec Králové , Charles University. Mothers of newly born infants who volunteered to participate did so after providing an informed consent. They received a small gift for their participation.

The experiment was administered in a quiet room at the maternity ward of the University Hospital in Hradec Králové . During the experiment, infants were asleep, lying supine in their cot (note that sleep does not seem to affect MMR in newborns, unlike in adults, Martynova et al., 2003). Auditory stimulation was through ER-3C earplugs (Etymotic research, Inc.), fitted in disposable earphones (Flexicouplers by Natus Europe, GmbH), at 67 dB SPL. If during the experiment an infant showed signs of waking up, the mother, who was present in the room throughout, was asked to calm them back to sleep. If an infant did not sleep, the experiment was terminated (this happened for 3 infants).

### 2.4 EEG recording and ERP analysis

**Fig. 2:**
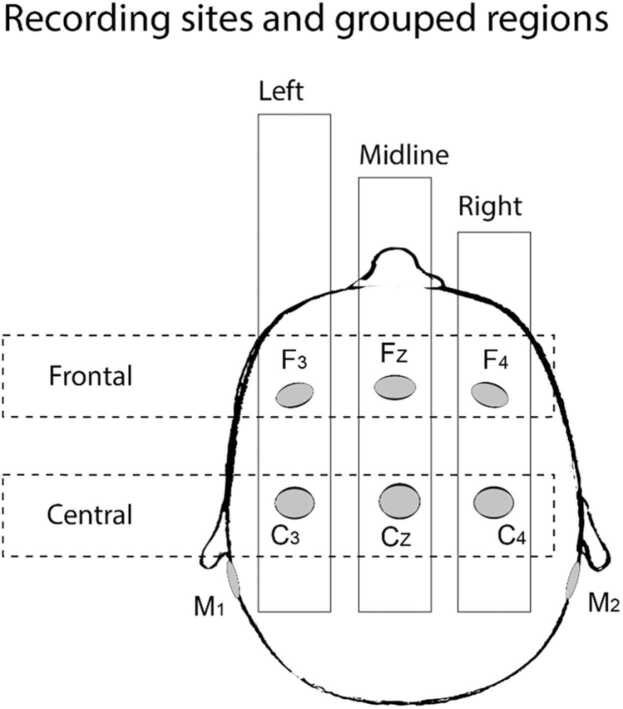
The recording sites and grouping of channels into 5 regions.

The EEG was recorded from six cephalic Ag/AgCl electrodes F3, FZ, F4, C3, CZ, C4 referenced to an electrode placed on the nose. Fig. 2 shows electrode locations and their grouping into regions that were used in statistical analyses. The signal amplifier had a bandwidth of 0.3–100 Hz (DEYMED Diagnostic s.r.o., Czech Republic). The EEG was recorded at a 3000-Hz sampling rate.

The data were processed with Matlab release 2019b (Mathworks, USA). In the recorded EEG, the frequencies above 40.0 Hz were removed using a digital filter (implemented in EEGLab, Delorme and Makeig, 2004). Therefore, the spectral content of the analyzed EEG was 0.3–40.0 Hz. The EEG signal was downsampled to 300 Hz and epoched. The epoch started 100 ms before and ended 1000 ms after the vowel or tone onset; mean voltage of the prestimulus part (from −100 ms to 0 ms) was subtracted from every epoch. The individual ERPs were calculated as an average of epochs with absolute amplitude under 90 µV. This procedure rejected about 25% of epochs; Table 2 shows the average number and the range of preserved epochs pooled across infants and channels. The level of signal to noise ratio for sensory ERP was determined by plus/minus procedure (Schimmel, 1967). We rejected 38 (out of 240) ERPs with SNR lower than one from further processing. The ERPs were additionally digitally filtered off-line by a low-pass Savitzky-Golay filter (Press et al., 1992, first polynomial order, window of 21 samples) to make responses better readable.

**Table 2:** Average count, minimum, and maximum of preserved epochs, pooled across infants and channels, for each stimulus type in the ERP and MMR analyses.

			ERPs			MMR: Deviants			MMR: Standards		
Domain	Presentation block	Stimulus	min	mean	max	min	mean	max	min	mean	max
speech	spectrum	ɛ	188	388	505	38	77	101	81	155	201
		a	181	382	499	35	77	100	67	153	200
	duration	ɛ	173	396	502	30	79	100	79	157	199
		ɛː	189	390	489	32	78	98	69	156	198
nonspeech	spectrum	ɛ	180	393	495	38	78	99	68	156	198
		a	208	387	495	36	78	99	82	155	199
	duration	ɛ	185	380	492	38	76	100	72	151	198
		ɛː	201	375	486	31	75	99	77	150	196

### 2.5 Statistical models

Data were analyzed with linear mixed-effects models using the packages *lmer*() and *lmerTest*() in R (Bates et al., 2015, Kuznetsova et al., 2017, R Core Team, 2016). One model was fitted for onset ERP, one for offset ERP, one for early MMR, and one for late MMR. The data entered in the model were ERP or MMR amplitudes averaged across trials per infant, dimension, electrode/scalp region, and stimulus type. The fixed and random-effects structures of each model are described in the respective Results subsections. In case of significant interactions, comparisons of the estimated 95% and 90% confidence intervals were done to localize the effect.

## 3 Results

### 3.1 ERPs: neural processing of stimulus physical properties

**Fig. 3:**
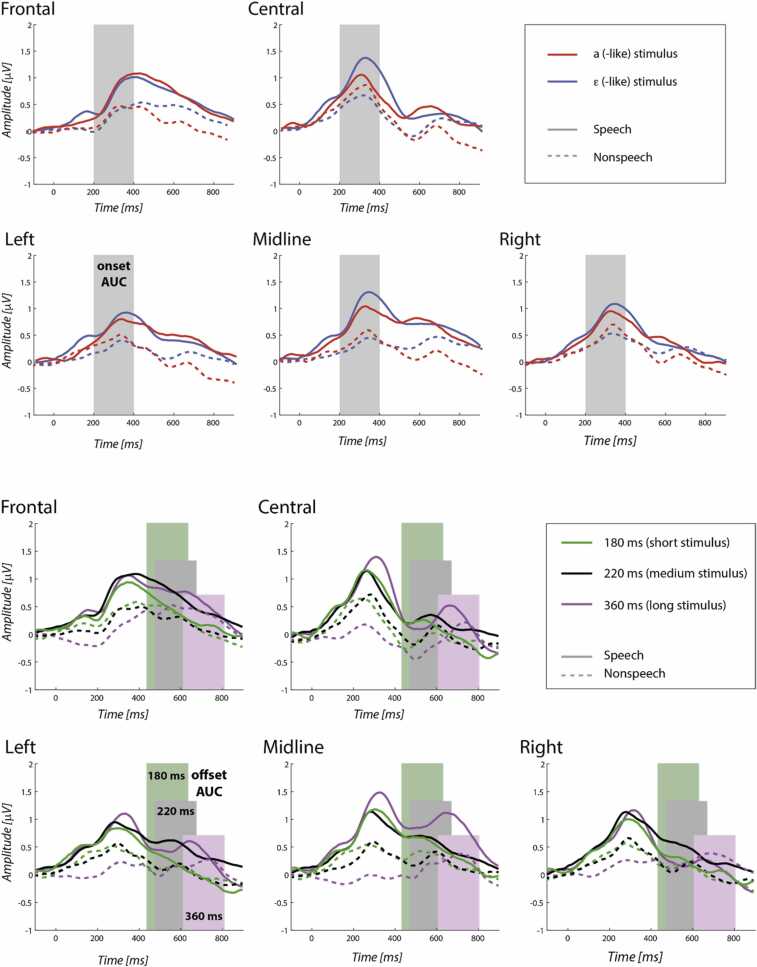
Upper five graphs: grand average ERPs to [a](-like) and [ɛ]/[ɛː](-like) stimuli. Lower five graphs: grand average ERPs to long, medium, and short stimuli. Shaded rectangles mark the analysis windows.

To test whether infants adequately processed the acoustic difference between the physically distinct stimuli, we compared the ERPs elicited by the acoustically different stimuli, i.e. averaging across all identical tokens with the exception of the first stimulus in each roving series. The ERPs were assessed in two 200-ms windows: an onset window 200–400 ms after vowel or tone onset, and an offset window 250–450 ms after vowel or tone offset. The window latencies were based on visual inspection of the grand-average waveforms, whereby the largest peak after stimulus onset was identified to lie at about 300 ms post-onset; and the largest peak after stimulus offset at about 350 ms after vowel or tone offset. The onset windows were aligned to vowel or tone onsets (i.e. the onset window in the speech stimulus was the onset of the V segment in the CV syllable) and were compared across stimuli that varied in their spectral properties. The offset windows were aligned to vowel and tone offsets and were as follows: 470–670 ms after stimulus onset for both the (medium-long) [a] and [ɛ] stimuli, 430–630 ms after onset for the short [ɛ] stimuli, and 610–810 ms after onset for the long [ɛː] stimuli. Offset responses were compared both across stimuli that varied in spectrum and across stimuli that varied in duration. The onset and offset responses were computed from ERP waveforms averaged across trials per infant, stimulus type, and electrode location, as areas under curve (AUC, in μV * ms) and submitted to the linear mixed models. The grand average ERPs are plotted in Fig. 3.

For the onset ERPs the model estimated the following parameters: the main effects of Domain (speech vs. nonspeech, coded as −0.5 vs. +0.5) and Spectrum ([a] vs. [ɛ(ː)] including the short, intermediate and long variants of [ɛ], coded as −0.5 vs. +0.5) and their interaction, the main effects of three location parameters, namely, Anteriority (central vs. frontal, coded as −0.5 vs. +0.5), Laterality (with two contrasts, namely, left and right vs. midline, coded as −0.25 and −0.25 vs. +0.5, and left vs. right, coded as −0.5 vs. +0.5), and their respective two- and three -way interactions with Domain and Spectrum. The model fitted per-participant random intercepts and random slopes for Spectrum. The offset model had the same predictors and random effects as the onset model, with main (fixed and random) and interaction effects of an additional parameter Duration (median-centred, coding 360 ms, 220 ms, and 180 ms, as +1, −0.2, and −0.6, respectively).

The fixed-effects outputs are given in Table 3. In both models, the intercept was reliably larger than zero, indicating that overall, there was a meaningful, positive-going response after both stimulus onset and offset, averaging to AUC of 124 μV*ms and 58 μV*ms, respectively. Both the onset and the offset response were affected by Domain: speech stimuli yielded larger onset and offset responses than nonspeech stimuli. Also, for both the onset and offset ERP, there were main effects of Laterality and Anteriority, but as these do not address any of our research questions we do not discuss them further.

**Table 3:** Fixed-effects output of the linear mixed models for the onset and offset ERP. Bold font marks effects with *p* below 0.05.

Parameter	Onset ERP					Offset ERP				
	Estimate	SE	df	t	p	Estimate	SE	df	t	p
Intercept	**123.876**	**11.711**	**109.157**	**10.578**	**<0.001**	**57.683**	**13.459**	**107.939**	**4.286**	**<0.001**
Domain (-speech, +nonspeech)	**-89.014**	**23.422**	**109.157**	**-3.800**	**<0.001**	**-58.24**	**26.918**	**107.939**	**-2.164**	**0.033**
Spectrum (-a, +e)	1.683	20.524	104.609	0.082	0.935	17.471	21.090	103.510	0.828	0.409
LateralityA (-lateral, +midline)	10.351	14.406	2204.536	0.718	0.473	**60.892**	**15.597**	**2104.588**	**3.904**	**<0.001**
LateralityB (-left, + right)	**25.425**	**12.476**	**2204.536**	**2.038**	**0.042**	1.907	13.508	2104.588	0.141	0.888
Anteriority (-central, +frontal)	**-30.073**	**10.187**	**2204.536**	**-2.952**	**0.003**	**59.474**	**11.029**	**2104.588**	**5.393**	**<0.001**
Domain * Spectrum	-45.120	41.048	104.609	-1.099	0.274	64.027	42.180	103.510	1.518	0.132
Domain * LateralityA	-54.618	28.812	2204.536	-1.896	0.058	-56.877	31.195	2104.588	-1.823	0.068
Domain * LateralityB	-9.511	24.952	2204.536	-0.381	0.703	36.330	27.016	2104.588	1.345	0.179
Domain * Anteriority	-13.940	20.373	2204.536	-0.684	0.494	-8.420	22.058	2104.588	-0.382	0.703
Spectrum * LateralityA	14.609	28.812	2204.536	0.507	0.612	14.957	31.632	2104.588	0.473	0.636
Spectrum * LateralityB	3.683	24.952	2204.536	0.148	0.883	5.826	27.394	2104.588	0.213	0.832
Spectrum * Anteriority	-30.501	20.373	2204.536	-1.497	0.135	10.144	22.367	2104.588	0.454	0.650
Domain * Spectrum * LateralityA	-29.405	57.625	2204.536	-0.510	0.610	44.325	63.264	2104.588	0.701	0.484
Domain * Spectrum * LateralityB	-6.750	49.904	2204.536	-0.135	0.892	-27.457	54.788	2104.588	-0.501	0.616
Domain * Spectrum * Anteriority	**101.350**	**40.747**	**2204.536**	**2.487**	**0.013**	10.955	44.734	2104.588	0.245	0.807
Duration						4.780	14.877	100.361	0.321	0.749
Domain * Duration						-1.775	29.754	100.361	-0.060	0.953
Duration * LateralityA						22.193	23.016	2104.588	0.964	0.335
Duration * LateralityB						-8.201	19.932	2104.588	-0.411	0.681
Duration * Anteriority						-20.748	16.275	2104.588	-1.275	0.203
Domain * Duration * LateralityA						**-121.303**	**46.031**	**2104.588**	**-2.635**	**0.008**
Domain * Duration * LateralityB						50.663	39.864	2104.588	1.271	0.204
Domain * Duration * Anteriority						1.429	32.549	2104.588	0.044	0.965

**Fig. 4:**
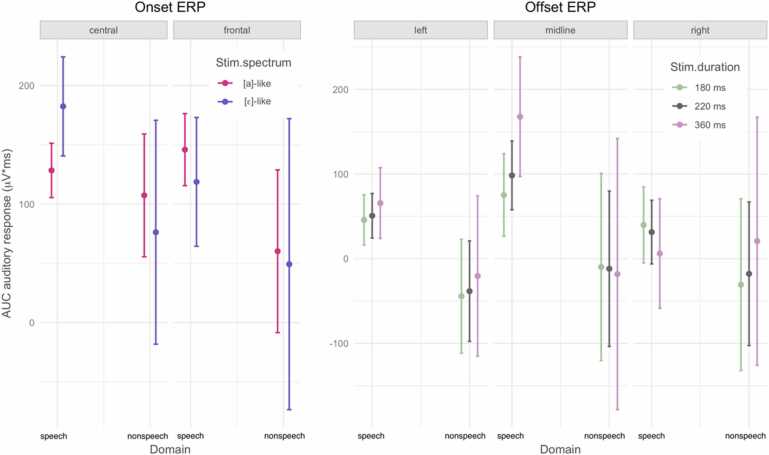
Modelled means and 95% CIs for the onset ERP (left) depicting the interaction of Domain, Spectrum, and Anteriority, and for the offset ERP (right) depicting the interaction of Domain, Duration, and Laterality. Colour coding aligns with the colours of the grand average ERP waves plotted in Fig. 3.

More importantly for the present questions, there were significant three-way interactions involving Domain. For the onset response, Domain interacted with Spectrum and Anteriority. Table 4 lists the means and standard errors of the modelled means for each stimulus type in each condition for the onset and offset ERP; Fig. 4 depicts the means along with their confidence intervals. The left-hand graph in Fig. 4 shows that the [ɛ/ɛː] speech stimuli yielded larger response than the [a] speech stimuli (while no such differences were detected in nonspeech), in the central region. For the offset response, Domain interacted with Duration and Laterality. The right-hand graph in Fig. 4 shows that on the midline channels, longer speech stimuli yielded a larger offset response than shorter speech stimuli, while no such effect was seen in the nonspeech stimuli or on the lateral channels.

**Table 4:** Modelled means and standard errors (SE) for onset ERP in the central and frontal region, and for offset ERP in the left, midline, and right region. Significance of pairwise comparisons (p.c.) across Stimulus types is indicated by asterisks: ** marks mutually exclusive means in the 95% confidence intervals estimated for each deviant type, * marks mutually exclusive means in 90% confidence intervals. Calculation of confidence intervals: 95% c.i. = mean ± 1.96SE, 90% c.i. = mean ± 1.645SE. The means and SEs were estimated using the *ggeffects* R package (Lüdecke, 2018, function ggpredict()).

	Onset ERP							Offset ERP									
Region →		central			frontal				left			midline			right		
Domain	Stimulus	mean	SE	p.c.	mean	SE	p.c.	Stimulus	mean	SE	p.c.	mean	SE	p.c.	mean	SE	p.c.
speech	a	128.443	11.711	**	145.9	15.5		short	45.7	15.2		75.2	24.9	**(lo.-sh.) *(lo.-me.)	39.8	22.9	
	ɛ	182.417	21.319		118.7	27.7		medium	50.7	13.4		98.3	20.7		31.4	19.2	
								long	65.6	21.3		167.7	36		6.1	33	
nonspeech	a	107.3	26.4		60.2	35		short	-44.4	34.3		-9.8	56.3		-30.6	51.7	
	ɛ	76.2	48.2		49.3	62.7		medium	-38.4	30.3		-11.9	46.8		-17.8	43.3	
								long	-20.4	48.3		-18.3	81.7		20.7	74.8

### 3.2 MMR: neural encoding of stimulus category

In order to test infants’ mental encoding of sounds across domains we compared their neural responses to identical stimuli in different functional contexts. Difference waves were calculated by subtracting the ERP elicited by a stimulus when it served as a standard (namely, the last two tokens in a row of 4–8 identical stimuli) from the ERP elicited by the same physical stimulus when it served as a deviant (namely, the first token in the row). These difference waves allowed us to quantify abstract processing of the stimuli beyond their physical properties, i.e. to assess whether and to what extent a physically identical stimulus was processed specifically to the functional/sequential context in which it occurred (i.e. fulfilling the role of a standard versus a deviant). We computed the AUC of the difference wave in two time windows whose latencies were based on visual inspection of the grand-averaged data and are in line with the early and late MMR windows used in previous studies: an early MMR 80–220 ms after change onset, and a late MMR 500–700 ms after change onset. ‘Change onset’ corresponded to vowel and tone onset in the spectral domain, and to the short vowel and short tone offset in the durational domain. To increase the signal to noise ratio (which, compared to the primary ERPs became low due to a lower number of epochs averaged), we pooled central and frontal channels sharing laterality (i.e. F3 & C3, Fz & Cz, and F4 & C4).

**Fig. 5:**
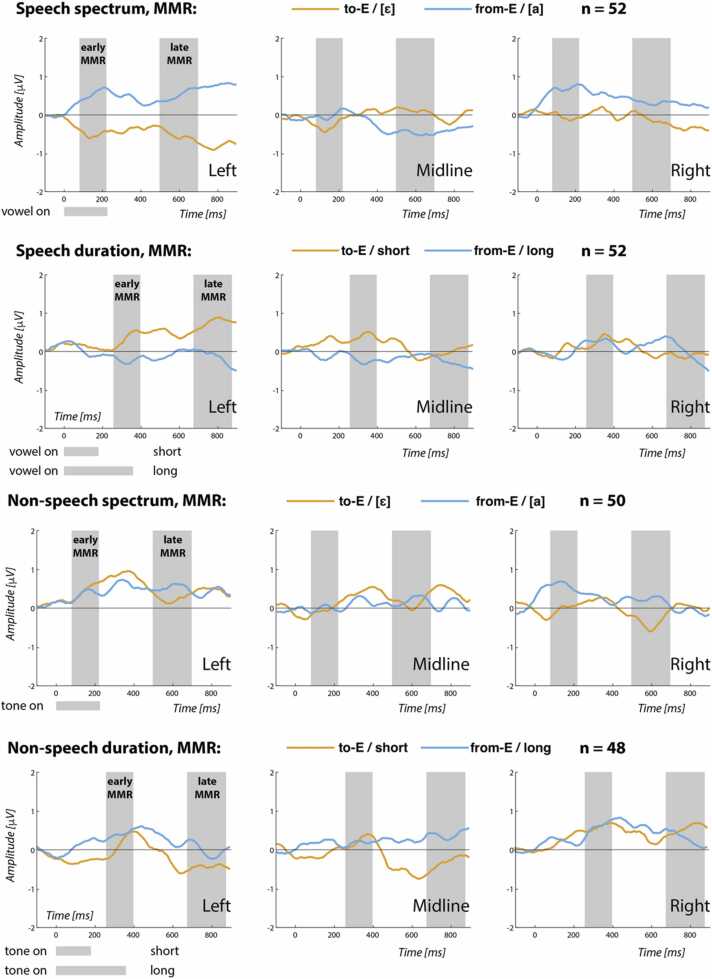
Grand average difference waves in the three scalp regions (for region visualization, see Fig. 2). Shading shows the early and late MMR analysis windows. Numbers in the top right corners show over how many participants averaging was done in each condition. The difference waves were computed from physically identical stimuli, e.g. the difference wave for the spectral “from-E” deviant was computed as: ERP for [a] as deviant minus ERP for [a] as standard, and the difference wave for the spectral “to-E” deviant was computed as: ERP for [ɛ] as deviant minus ERP for [ɛ] as standard, and likewise for the durational deviations between [ɛ] and [ɛː].

Deviant identities were coded as follows. The spectral deviation from [fa] to [fɛ] (and alike for nonspeech stimuli) was coded as a change “to E” and the spectral deviation from [fɛ] to [fa] as a change “from E”; and alike for the nonspeech stimuli. Similar coding was adopted for deviant changes on the durational dimension, such that the durational deviation from [fɛː] to [fɛ] was coded as a change “to E”, and the durational deviation from [fɛ] to [fɛː] was coded as a change “from E”; and alike for the nonspeech stimuli. Fig. 5 plots the grand average difference waves.

Linear mixed effects models estimated the main effects of Domain (speech vs. nonspeech, coded as −0.5 vs. +0.5), Dimension (duration vs. spectrum, coded as −0.5 vs. +0.5), Deviant (to-E vs. from-E, coded as −0.5 vs. +0.5), and all their two- and three-way interactions. The MMR models also included the main effect of Laterality (with two contrasts, namely, left and right vs. midline, coded as −0.25 and −0.25 vs. +0.5, and left vs. right, coded as −0.5 vs. +0.5) and its respective two-, three-, and four-way interactions with Domain, Dimension, and Deviant. The models included per-participant random intercepts and random slopes for Dimension and Deviant, and their interaction.

Table 5 lists the output. For the early MMR, the first Laterality contrast turned out significant showing that the amplitude of the early MMR was smaller on the midline than laterally. For the late MMR, there was a significant three-way interaction of Domain, Dimension, and Laterality as well as a significant three-way interaction of Dimension, Deviant, and Laterality, both of which are licenced by a significant higher-order interaction.

**Table 5:** Fixed-effects output of the linear mixed models for the early and the late MMR. Bold font marks effects with *p* below 0.05.

Parameter	Early MMR					Late MMR				
	Estimate	SE	df	t	p	Estimate	SE	df	t	p
Intercept	26.916	16.448	90.652	1.636	0.105	8.168	32.397	100.224	0.252	0.802
Domain (-speech +nonspeech)	28.050	32.896	90.652	0.853	0.396	23.630	64.794	100.224	0.365	0.716
Dimension (-duration +spectrum)	-13.848	31.791	90.342	-0.436	0.664	8.855	71.592	105.082	0.124	0.902
Deviant (-toE +fromE)	18.690	32.537	106.125	0.574	0.567	29.245	54.834	101.250	0.533	0.595
LateralityA (-lateral +midline)	**-37.640**	**17.794**	**2020.00**	**-2.115**	**0.035**	-24.677	34.790	2020.000	-0.709	0.478
LateralityB (-left +right)	28.404	15.410	2020.00	1.843	0.065	1.532	30.129	2020.000	0.051	0.960
Domain * Dimension	-24.203	63.581	90.342	-0.381	0.704	23.321	143.184	105.082	0.163	0.871
Domain * Deviant	1.860	65.075	106.125	0.029	0.977	65.264	109.669	101.250	0.595	0.553
Dimension * Deviant	88.837	69.387	105.271	1.280	0.203	65.800	113.763	102.553	0.578	0.564
Domain * LateralityA	-13.235	35.587	2020.00	-0.372	0.710	62.340	69.580	2020.000	0.896	0.370
Domain * LateralityB	-28.206	30.819	2020.00	-0.915	0.360	37.686	60.258	2020.000	0.625	0.532
Dimension * LateralityA	-18.029	35.587	2020.00	-0.507	0.613	48.439	69.580	2020.000	0.696	0.486
Dimension * LateralityB	-34.191	30.819	2020.00	-1.109	0.267	-76.024	60.258	2020.000	-1.262	0.207
Deviant * LateralityA	-65.521	35.587	2020.00	-1.841	0.066	-65.846	69.580	2020.000	-0.946	0.344
Deviant * LateralityB	25.909	30.819	2020.00	0.841	0.401	0.883	60.258	2020.000	0.015	0.988
Domain * Dimension * Deviant	-148.284	138.775	105.271	-1.069	0.288	-128.812	227.526	102.553	-0.566	0.573
Domain * Dimension * LateralityA	27.083	71.175	2020.00	0.381	0.704	29.797	139.160	2020.000	0.214	0.831
Domain * Dimension * LateralityB	-21.371	61.639	2020.00	-0.347	0.729	**-248.684**	**120.516**	**2020.000**	**-2.063**	**0.039**
Domain * Deviant * LateralityA	52.623	71.175	2020.00	0.739	0.460	192.463	139.160	2020.000	1.383	0.167
Domain * Deviant * LateralityB	7.167	61.639	2020.00	0.116	0.907	-73.560	120.516	2020.000	-0.610	0.542
Dimension * Deviant * LateralityA	-26.879	71.175	2020.00	-0.378	0.706	**-328.215**	**139.160**	**2020.000**	**-2.359**	**0.018**
Dimension * Deviant * LateralityB	6.090	61.639	2020.00	0.099	0.921	-66.909	120.516	2020.000	-0.555	0.579
Domain * Dimension * Deviant * LatA	37.529	142.349	2020.00	0.264	0.792	120.722	278.320	2020.000	0.434	0.665
Domain * Dimension * Deviant * LatB	**261.845**	**123.278**	**2020.00**	**2.124**	**0.034**	**507.961**	**241.033**	**2020.000**	**2.107**	**0.035**

**Fig. 6:**
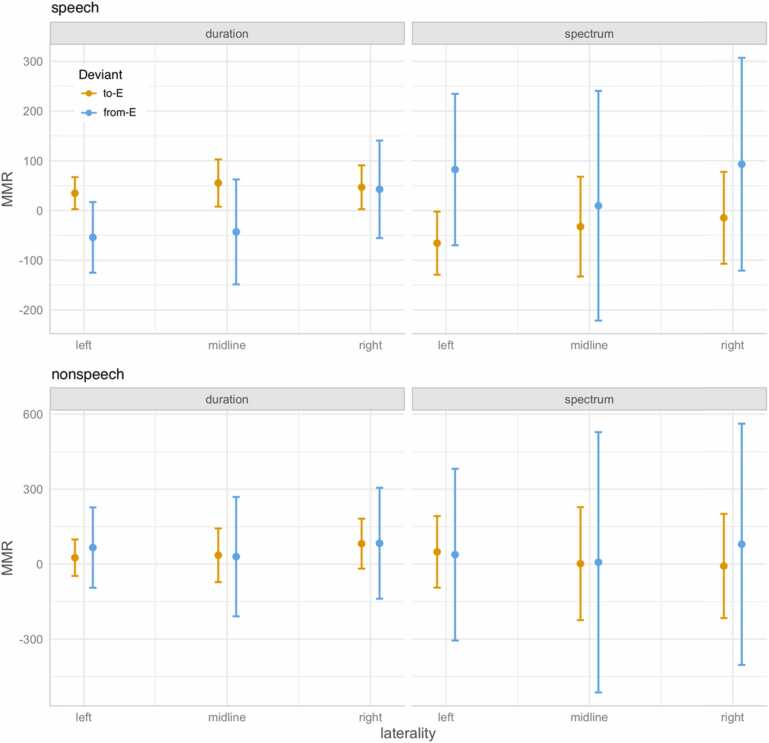
The modelled means and 95% c.i.s for the early MMR, unpacking the Domain * Dimension * Deviant * Laterality interaction. Deviant label “to-E” corresponds to the [ɛ] deviant on the spectral dimension and to the short deviant on the duration dimension, and deviant label “from-E” corresponds to the [a] deviant on the spectral dimension and to the long deviant on the duration dimension.

The four-way interaction of Domain, Dimension, Deviant, and Laterality (left vs right) turned out significant for both the early and the late MMR. To unpack the interaction, we inspected the modelled means and compared them across the two Deviants in all conditions; Fig. 6 plots the means and 95% confidence intervals for the early MMR and Table 6 lists the means and standard errors for both the early and the late MMR. The pairwise comparisons show that in the speech domain the from-E, i.e. long, duration deviant yields a more negative MMR than the to-E, i.e. short, duration deviant on the left hemisphere (comparison of 95% c.i.s) and on the midline (comparison of 90% c.i.s). In the speech domain but this time on the spectral dimension, the to-E, i.e. [ɛ], spectral deviant yields a more negative MMR than the from-E, i.e. [a], spectral deviant (comparison of 90% c.i.s). Interestingly, the entire 95% c.i. of the [ɛ] spectral deviant on the left hemisphere is below zero, i.e. is reliably negative, arguably indexing a (relatively) mature MMR response – this is the only condition that elicits a mismatch *negativity*, i.e. MMN. For the late MMR, only the durational condition in speech shows a significant directional asymmetry in the left hemisphere (comparison of 90% c.i.s).

**Table 6:** Modelled means and standard errors (SE) for early and late MMR at left, midline, and right channels. Significance of pairwise comparisons (p.c.) across Deviants is indicated by asterisks: ** marks mutually exclusive means in the 95% confidence intervals estimated for each deviant type, * marks mutually exclusive means in 90% confidence intervals. Calculation of confidence intervals: 95% c.i. = mean ± 1.96SE, 90% c.i. = mean ± 1.645SE. The means and SEs were estimated using the *ggeffects* R package (Lüdecke, 2018, function ggpredict()).

				Early MMR									Late MMR								
Laterality →				left			midline			right			left			midline			right		
Domain	Dimension	Deviant		mean	SE	p.c.	mean	SE	p.c.	mean	SE	p.c.	mean	SE	p.c.	mean	SE	p.c.	mean	SE	p.c.
speech	duration	to-E	[short]	34.9	16.4	**	55.3	24.2	*	46.8	22.5		124.4	32.4	**	-12.4	47.5		-16.1	44.2	
		from-E	[long]	-54.0	36.2		-43.0	53.8		42.7	50.0		-51.2	67.0		-64.7	102.7		6.4	95.0	
	spectrum	to-E	[ɛ]	-65.6	32.4	*	-32.3	51.2		-14.8	47.2		-104.8	69.6		33.6	104.2		-36.6	96.7	
		from-E	[a]	82.3	77.7		9.6	117.8		93.0	109.2		107.4	153.4		-82.8	231.4		52.9	214.6	
non-speech	duration	to-E	[short]	25.8	37.2		35.6	54.8		82.0	51.0		-86.7	73.4		-74.5	107.6		98.6	100.2	
		from-E	[long]	66.3	82.1		30.3	121.8		83.6	113.2		-1.9	151.7		68.8	232.4		54.0	215.0	
	spectrum	to-E	[ɛ]	49.3	73.0		2.0	115.4		-7.9	106.4		30.8	157.2		43.9	235.0		-77.8	218.2	
		from-E	[a]	38.1	175.3		7.4	265.8		78.9	246.3		90.4	346.0		54.7	521.8		39.4	483.9	

## 4 Discussion

### 4.1 Primary ERP responses

Hearing simple consonant-vowel syllables or inharmonic tone complexes elicited an automatic sensory response in newborns’ brains. This means that newborn infants neurally process auditory stimuli both when they are speech and when they are nonspeech. Furthermore, the sensory responses elicited by the vowels were larger than those elicited by the complex tones. This indicates specialized cortical tuning to speech at the very level of its basic building blocks, and further extends the earlier documented infants’ preferences for listening to larger chunks of speech versus analogue non-speech stimuli. Also, these automatic sensory responses elicited in sleep demonstrate a neural parallel to the earlier found behavioural preferences for speech over nonspeech in awake newborns’ (Vouloumanos and Werker, 2007).

As evidenced by the triple interactions involving Domain for the onset and the offset ERP responses, the spectral difference between [a] and [ɛ] was reflected in significantly different onset ERP responses to [a] versus [ɛ] in the speech condition at central channels, and the durational difference between short [ɛ] and long [ɛː] was reflected in different offset ERPs to short versus long vowels at midline. This means that besides speech eliciting stronger neural responses than nonspeech in general, the *acoustic differences* between stimuli in terms of the first three formants, as well as in terms of duration, were more accurately processed when the stimuli were speech and less so when they were nonspeech. The more distinct acoustic response to the formant and duration differences in speech might be explained by a finer (experience-based or innate) cortical tuning to speech. Alternatively, the more accurate processing in speech could be due to differential stimulus complexity across our stimulus sets. The speech stimuli were spectrally richer such that higher formants above F3 (which were not present in the non-speech condition) could have contributed to the perceived difference between [a] and [ɛ]. In a similar fashion, the fact that the vowels were preceded by a fricative consonant of constant duration might have facilitated the processing of the duration difference between short [ɛ] and long [ɛː] as compared to the short and long tones presented in isolation.

The topographical distribution of the auditory responses across the two domains, with midline and central regions reflecting robust processing of the acoustic content of linguistic stimuli, suggests a somewhat integrated processing pathway for speech. Thus, not only do speech stimuli differ from nonspeech analogues in that they are processed more robustly overall, but they also seem to activate other neural populations, whose specialisation remains to be determined.

### 4.2 Mismatch responses

The mismatch responses (MMR) patterned differently for speech than for nonspeech. The processing of speech sounds was asymmetrical: left-laterally, the [a] to [ɛ] change resulted in a more negative response than the [ɛ] to [a] change (and the [a] to [ɛ] change in speech was also the only condition that brought about a reliably negative MMN), and the [ɛ] to [ɛː] change resulted in a more negative response than the [ɛː] to [ɛ] change (and this durational asymmetry was observed also on the midline). Our first prediction that speech stimuli, unlike nonspeech, will yield a more mature MMR response is thus, partially, borne out. As the directional, left-lateralized asymmetries occurred both for the spectral and for the durational dimension in speech, our second prediction about vowel length having a developmental precedence over vowel quality is not supported.

The lateralization of the speech processing asymmetries to the left hemisphere adds to previous literature on hemispheric specialization for speech. Studies on the neural development of phoneme processing suggest that segmental speech processing starts bilaterally and only after the sixth month of an infant’s development comes to be left-lateralized to resemble the hemispheric specialization found in adults (Arimitsu et al., 2011, Sato et al., 2012), although there are indications of left-hemisphere advantage in much younger infants (Dehaene-Lambertz and Baillet, 1998). Neurolinguistic studies with infants typically do not examine directionality effects in speech sound processing and therefore any subtle lateralization effects (corresponding to maturation) might have been previously obscured. Further work, with e.g. multichannel EEG that enables to more accurately localize sources of neural activity, is needed to confirm (or disprove) the lateralization of directional asymmetries detected here.

The newborns’ left-lateralized asymmetries between the vowel quality deviants are reminiscent of the asymmetries previously reported for adults in some languages (e.g., Lahiri and Reetz, 2010, but see Mitterer, 2011, for counterevidence). Recall that in the present experiment, a change from [fa] to [fɛ] elicited a more robust negative mismatch response than a change from [fɛ] to [fa]. Although for instance German adults sometimes show similar directional effects for comparable vowel contrasts (e.g. Scharinger et al., 2012), Czech adults’ neural discrimination of [fa]-[fɛ] exhibits an asymmetry in the opposite direction (Nudga et al., 2021). According to the Featurally Underspecified Lexicon (FUL, Eulitz and Lahiri, 2004) the specificity of speech sounds’ mental representations determines whether and how much a sound is predictive, i.e. whether and how much its replacement by another speech sound violates a listener’s expectation and causes an MMN. Assuming acquired, i.e. language-specific, phonological representations, Nudga et al., 2021 argued that Czech /a/ is phonologically underspecified (for backness), causing that a change from the un(der)specified, less predictive /a/ to a fully-specified /ɛ/ does not violate an expectation in Czech adult listeners while a reverse change does. The Czech newborns in the present study had an MMN asymmetry in the opposite direction, which indicates that their processing – quite expectedly – was *not* affected by the phonological makeup of the Czech vowel system.

Although lacking phonological knowledge, newborns do have some prior experience with speech in terms of its acoustics. An account that addresses asymmetries shaped by phonetic biases in young infants has been proposed by Polka and Bohn, 2003, Polka and Bohn, 2011. These authors’ Natural Referent Vowel framework refers to vowels’ articulatory-acoustic properties and argues that peripheral vowels such as [a], [i], and [u], thanks to their unique articulatory-acoustic characteristics, are stable points in the vowel space and universally serve as perceptual anchors. Other authors (Schwartz et al., 2005) argued that it is the acoustic properties of peripheral vowels, namely the closeness of neighbouring vowel formants, i.e., focalization, which makes vowels like [a], [i], and [u] perceptually prominent. According to the NRV (Polka and Bohn, 2011), a young infant who has been exposed to spoken language will discriminate a change from a nonperipheral [ɛ] to a peripheral [a] more robustly than a change in the reverse direction (this directionality effect has been confirmed in the meta-analysis by Tsuji and Cristia, 2017), while later in development these auditorily-conditioned asymmetries may leave way for language-specific patterns (Pons et al., 2012; but see Tsuji and Cristia, 2017, who did not find an interaction effect of age and nativeness). The asymmetry detected in the present experiment with newborns is not in line with the asymmetry predicted by the NRV.

We propose that the perceptual asymmetry in our newborn data might be caused by differential learning stages for each of the two vowel categories. The concentrated energy at about 1 kHz – which is a frequency band that still has a relatively good chance of propagating into the womb (Richards et al., 1992) – makes [a] perceptually more salient (and especially so *in utero*) than [ɛ] whose energy is dispersed across a wider frequency range*.* Furthermore, in spoken Czech tokens of /a/ are more frequent than tokens of /ɛ/ (by about 15–20%, ORAL v1, 2019). Hypothetically, fetuses who had been exposed to somewhat vaguely audible and slightly less frequent [ɛ]s and to better audible and more frequent [a]s, could have more readily started to form a perceptual category for /a/ than for /ɛ/. Upon hearing tokens of [a] after birth (in the present experiment), the neonate listeners recognized a previously encountered, and perhaps somewhat ‘primitively’ learned /a/-category, and could establish a memory trace for it during the experimental paradigm such that with every upcoming trial they anticipated hearing that vowel category (in line with the predictive coding theory, see Winkler and Czigler, 2012). When the [a] stimulus changed into [ɛ], their memory trace of /a/ was violated, as reflected in a strong MMN response to the [ɛ] deviant. On the contrary, upon hearing tokens of [ɛ], there was no category to be recognized, no memory trace was built up during a repeated presentation of [ɛ]s, such that a change from [ɛ] to [a] did not violate any expectation. This is why the [a] deviant resulted in a much weaker MMR than the [ɛ] deviant.

As a reviewer pointed out, phonetic warping-induced asymmetries are addressed by the Native Language Magnet model (NLM, Kuhl, 1991; Kuhl et al., 2008). According to the NLM, the internal structure of segmental speech categories (acquired through exposure), which comprises a best instance of the category - the prototype, and its variants, predicts directional asymmetries. The prototype acts as a perceptual magnet: when the prototype is heard first, the difference between it and a non-prototypical variant is perceived as smaller than when the variant is heard first. Even though the present experiment tested discrimination *across two* adult categories, one could potentially argue that the fetuses/newborns would warp the entire vowel space of [a]s and [ɛ]s into a single ‘protocategory’ (as also modelled by Chládková et al., 2020). Assuming such a protocategory in which the focal and more frequent [a] is more prototypical than the less salient and less frequent [ɛ], the NLM would predict better discrimination for a change from [ɛ] to [a] than vice versa, which is the opposite of what we found in the newborns’ MMR. At this point, it is unclear whether the newborns perceived [a] and [ɛ] as instances of one protocategory, or as two different – and perhaps differently well-warped – adult categories, or whether they were still blank-slates without any prior warping/categorization having occurred.

Although neither of the two influential models of early speech perception, the NRV and the NLM, did specifically refer to prenatal development, it is intriguing that the asymmetries we detected here with newborns run counter to both the phonetically-based NRV’s as well as the categorization-based NLM’s predictions. Potentially, the language-general biases predicted by the NRV (Polka and Bohn, 2011), or the prototype-driven biases predicted by the NLM (Kuhl et al., 2008), might occur in slightly older infants after sufficient experience with speech *ex utero*, or, they might, after all, be language- or phoneme-specific (i.e. not applicable to infants acquiring Czech, or to [mid-]low vowels such as [ɛ] and [a]).

Could the present reversal of NRV- or NLM-predicted asymmetries be attributed to having measured discrimination at the neural level? The NRV was proposed to explain asymmetries found in infants’ behavioral discrimination (Polka and Bohn, 2011), and the NLM’s predictions for asymmetries were, too, mostly attested with behavioral methods (e.g. Moon et al., 2013; but note that Kuhl et al., 2008, explicitly propose that exposure to native language will result in language-specific processing at the neural level). Neural discrimination patterns are typically – at least to some extent – reflected in behavioral measures of vowel discrimination (see the review in Näätänen, 2001, for early work and e.g. Virtala et al., 2018, Virtala et al., 2020 for more recent work). If anything, neural change detection *precedes* behavioral change detection: Tremblay et al. (1998) showed that after training the MMN to phoneme contrasts improved even though such improvement was not detectable at the level of behavior. As for the case of perceptual asymmetries, one may expect that neurally a contrast could yield a similarly strong MMN in both directions of change, yet behaviorally one direction would be discriminated more readily than the other direction (see Polka et al., 2021, who did not detect a MMN asymmetry for [y]-[u] in adults who typically have an asymmetry in behavioral tasks). A complete *reversal* of an asymmetry across the neural MMR and behavioral level would mean that a direction of change that is poorly detectable by a neural, pre-attentive, index of discrimination is well detectable behaviorally, which we consider rather unlikely. We thus like to argue that the dissonance between ours and NRV- or NLM-like asymmetries is not due to the use of MMR in the present experiment. Nevertheless, it is still worth exploring further whether measures of neural speech processing other than the MMR reveal (other kinds of) perceptual asymmetries: a potential measure to look at is the oscillatory theta or gamma activity. In infants theta activity seems to reflect general phonetic decoding of speech irrespective of comprehension, and gamma activity relates to processing of language-specific/syllabic information (Ortiz Barajas et al., 2021): in potential future work on infants’ perceptual asymmetries and neural oscillations one might hypothesize that phonetically-shaped biases be reflected in the theta band (which is also what Polka et al., 2021, observed in adults) and categorically-shaped biases in the gamma-band.

Let us now turn to the perceptual asymmetry in the durational vowel contrast. As far as contrasts such as /a/-/ɛ/ are concerned, the literature relatively widely documents and theorizes about the asymmetries. Much less is known about potential asymmetries in the perception of length. Previous studies, mostly with adults, typically (though not always) find that listeners more robustly process changes from short to long stimuli than vice versa, probably because an addition of information is more readily detectable than a loss of it (Jaramillo et al., 1999, Ylinen et al., 2006). The short-to-long easy detectability does not, however, explain the perceptual patterns of the newborns in the present study. There was a more negative MMR to a short-to-long deviant than to a long-to-short deviant in speech, but no such effect was seen in the non-speech stimuli which differed in duration in exactly the same way as the speech sounds. Therefore, the asymmetry in speech might not be (entirely) due to the immediate stimulus acoustic properties.

Although the NRV (Polka and Bohn, 2011) addresses vowel length only briefly, it suggests that short vowels may – similarly to focal vowels – serve as perceptual anchors, such that discriminating a change from a long to a short vowel would then be easier than vice versa. Regarding the prototype-biases postulated by the NLM (Kuhl et al., 2008), the more frequent short vowel could be considered more prototypical than the long one, thus predicting better discrimination from long to short than vice versa. The durational asymmetry that we found here is, again, a reversal of the asymmetry postulated by the NRV and the NLM frameworks.

As in the case of the spectral contrast, the MMR asymmetry for vowel length could possibly reflect the newborns’ prior experience with durationally varying *speech* input and differential degree of warping for the short versus the long categories. In Czech, short vowels are more frequent than long vowels (ORAL v1, 2019). Also, considering absolute duration scales, it appears that tokens of Czech short vowels are rather compactly clustered around a prototypical short value, while tokens of Czech long vowels are a bit more widely spread around a particular long duration value (Lehiste, 1970, Paillereau and Chládková, 2019), and this differential dispersion in the short and the long category might in prenatal IDS be even larger than in ADS (Chládková et al., 2019).^3^ A developing fetus who encounters many similarly short vowels and fewer variously long vowels might more readily recover and start warping the narrowly-defined underlying short category as opposed to a broadly-distributed underlying long category. In the current experiment, upon hearing tokens of the (partially) warped short category, the newborn listener might establish a memory trace and build up a prediction, which – when violated by a long stimulus – results in a more negative MMR response than does a reverse violation.

In this section we speculated about the possible cause of MMR asymmetries in vowel perception at birth. We argued that the newborns’ speech-specific left-lateralized asymmetries in neural discrimination of vowels may reflect a more advanced stage of perceptual warping for some vowel categories than for others. At this point however, we cannot rule out an alternative explanation that the perceptual patterns seen here are universal, innate, and have no relation to the language spoken in the babies’ environment. In that respect, the asymmetries could simply reflect infants’ general preference for, or tuning into, speech over nonspeech. To what extent prenatal experience with speech leads to early perceptual categorization of the ambient speech sounds remains to be tested.

### 4.3 Limitations and future research

A potential methodological confound to the speech vs. nonspeech sensory ERP comparison is the extent to which the material in each domain was informative. We aimed to present the same acoustic patterns in the context of speech and nonspeech stimuli. Since vowels hardly ever occur as isolated segments in natural speech, we used the smallest typically occurring speech units – consonant-vowel monosyllables. Besides strengthening the ‘speech-likeliness’ of the stimuli, the syllable-initial fricative might have, however, provided supporting acoustic information. The [f] had invariant duration and frication formant, which could have served as reference points for perceptual discrimination and categorization of the immediately following vowel. Potentially, the initial fricative might have contributed to the stronger primary ERPs to acoustic stimulus differences in speech as compared to nonspeech. (However, it is less likely that the fricative contributed to the asymmetries in MMR *within* the speech condition, since all speech stimuli began with an identical fricative.) To resolve whether the stronger primary responses to speech were domain-specific, or were driven by the extra acoustic information, a possible follow-up experiment could employ nonspeech stimuli that entail referencing information, roughly comparable to an initial consonant in CV syllables.

We proposed that prenatal experience with listening to speech could have resulted in the asymmetries observed in this study. To assess the plausibility of prenatal vowel learning, one needs to test infants, and/or near-term fetuses with different language backgrounds. However, those populations are particularly demanding to recruit and test (and especially so for a cross-language design), and have noisier data than older listeners. To that end, computational modelling may provide valuable insights, leading to informed hypotheses for experiments with such young humans. Seebach et al. (1994) tested whether the English plosive place of articulation is learnable prenatally. A neural network, modelling the fetal hearing capacities and intrauterine sound properties, was exposed to realizations of English /pa/-/ta/-/ka/. The network came to differentiate the three-way categorical contrast and even generalized the acquired knowledge to untrained /ba/-/da/-/ga/. One could thus hypothesize (and test) that near-term fetuses, exposed to English would perceptually discriminate (and perhaps even categorize) the three-way consonantal place distinction.

Using two-layer neural networks, research in our lab showed that Spanish but not Czech near-term virtual fetuses will form two separate ‘protocategories’ for [a]- and [ɛ]-like vowels (Chládková et al, 2020). In a cross-linguistic experiment, Spanish-exposed newborns would thus be predicted to discriminate [a] and [ɛ] more robustly than Czech-exposed newborns. Considering the present MMR asymmetries in Czech newborns’ processing of [fa]-[fɛ], a more robust discrimination by Spanish newborns might mean an overall more negative and/or symmetric MMR. Supposedly, fetuses and newborns exposed to Spanish, which, unlike Czech, does *not* contrast short and long vowels, might have an attenuated MMR to a vowel length distinction, such as the [fɛ]-[fɛː] used here. Alternatively, one could test a single language group of newborns on changes within- and across adult categories: Czech newborns could be tested on their neural discrimination of variants of /ɛ/ and variants of /a/. If prenatal phonetic warping takes place – perhaps for /a/ if it is focalization, or perhaps for /ɛ/ if it is the lowest formant that matters *in utero* – one could expect to find prototype-like directional effects in the strength of MMR for that particular vowel category (Kuhl et al., 2008). A cross-sectional study comparing newborns to older infants (e.g. 6- and 12-month olds) could help identify the degree of warping/categorization at birth (if any).

The present study does not answer the question of whether segmental speech sound learning starts already *in utero*: the hypothesized, input saliency-based, difference in newborns’ categorization of phonemic vowel length versus phonemic vowel quality was not found. However, the unexpected left-lateralized directional asymmetry of the newborns’ neural discrimination for both phoneme contrasts offers new insights into the earliest stages of speech learning: it has lead us to speculate about a potential scenario of prenatal speech development which is testable in future work. Ultimately, experiments that compare newborn infants or fetuses from different language environments are crucial in order to answer questions about the effects that prenatal experience has on the formation of speech sound categories in the young infant.

## 5 Conclusions

We pursued the question of whether humans might learn about the speech sounds of their language before they are born, and whether some speech categories are learned earlier than others. Sleeping newborns listened to native-language speech sound differences, namely, [fɛ]-[fa] and [fɛ]-[fɛː], and to similar nonspeech stimuli, namely, inharmonic complex tones.

Sensory ERPs to the speech stimuli were overall stronger and more reliably reflected the differences in stimulus spectral and durational characteristics than did the ERPs to nonspeech. The mismatch responses differed across domains, indicating left-lateralized directional asymmetries in the processing of speech stimuli. Contrary to our predictions, we did not detect any differences between the two types of speech contrasts, suggesting that phonemic vowel length and vowel spectral quality contrasts were, by the third day of life processed comparably.

The most intriguing result were the directional asymmetries in speech. Left-laterally, infants had a more mature mismatch response to a change from [fa] to [fɛ] than vice versa, and to a change from [fɛ] to [fɛː] than vice versa. We proposed a hypothetical scenario of how prior experience could modulate newborn speech sound processing, arguing that the newborns’ perceptual asymmetries reflected differential degrees of prenatal perceptual warping of /a/ versus /ɛ/, and of /ɛ/ versus /ɛː/. To what extent our proposal is realistic – and in general, whether naturalistic speech sound category formation occurs before birth – remains to be addressed in future work.

## Declaration of Competing Interest

The authors declare that they have no known competing financial interests or personal relationships that could have appeared to influence the work reported in this paper.

## Data Availability

[The data that support the findings of this study are available at OSF https://osf.io/b9txc/].

## Footnotes

1 In fact, as the MMR is an ERP difference, reporting sensory responses should be mandatory even in adult studies because the same measured MMR may result from different conditions. For example, no response to deviant and a small response to standard will create an difference response, which might be wrongly interpreted in the sense of the prediction error even though the neural system does not respond to one of the stimuli at all (see Kremláček et al., 2016).

2 The total sample size of 120 tested infants (60 per condition) was limited by a 3-month testing period in the hospital, and was considered sufficient to obtain clean data from about 50 infants for the speech and 50 infants for the nonspeech condition, counterbalancing the order of presentation of the durational and the spectral change block within each group. N ~ 50 per condition is roughly comparable to what is typically attested in the field of recent newborn ERP research (see e.g. Thiede et al., 2019).

3 However, when logarithmic scales for duration are considered, which are perhaps more psychoacoustically plausible than absolute scales (Abel, 1972), the dispersion in short and long vowel categories seems comparable.
